# Putting prevention into practice: qualitative study of factors that inhibit and promote preventive care by general practitioners, with a focus on elderly patients

**DOI:** 10.1186/1471-2296-11-68

**Published:** 2010-09-20

**Authors:** Ulla Walter, Uwe Flick, Anke Neuber, Claudia Fischer, Rugzan J Hussein, Friedrich W Schwartz

**Affiliations:** 1 Institute for Epidemiology, Social Medicine and Health System Research, Hannover Medical School, Carl-Neuberg Strasse 1, OE 5410, 30623, Hannover, Germany; 2 Alice Salomon University of Applied Sciences, Alice-Salomon-Platz 5, 12627, Berlin, Germany; 3 University of Kassel, Dekanat des Fachbereiches 04, Arnold-Bode-Strasse 10, D-34109 Kassel, Germany

## Abstract

**Background:**

General practitioners (GPs) have a key role in providing preventive care, particularly for elderly patients. However, various factors can inhibit or promote the implementation of preventive care. In the present study, we identified and examined factors that inhibit and promote preventive care by German GPs, particularly for elderly patients, and assessed changes in physicians' attitudes toward preventive care throughout their careers.

**Methods:**

A qualitative, explorative design was used to identify inhibitors and promoters of preventive care in German general medical practice. A total of 32 GPs in Berlin and Hannover were surveyed. Questions about factors that promote or inhibit implementation of preventive care and changes in physicians' perceptions of promoting and inhibiting factors throughout their careers were identified. Episodic interviews, which encouraged the reporting of anecdotes regarding daily knowledge and experiences, were analyzed using ATLAS/ti. Socio-demographic data of GPs and structural information about their offices were collected using short questionnaires. The factors identified as inhibitory or promoting were classified as being related to patients, physicians, or the healthcare system. The changes in GP attitudes toward preventive care throughout their careers were classified as personal transitions or as social and health policy transitions.

**Results:**

Most of the identified barriers to preventive care were related to patients, such as a lack of motivation for making lifestyle changes and a lack of willingness to pay for preventive interventions. In addition, the healthcare system seemed to inadequately promote preventive care, mainly due to poor reimbursement for preventive care and fragmentation of care. GPs own attitudes and health habits seemed to influence the implementation of preventive care. GPs recognized their own lack of awareness of effective preventive interventions, particularly for elderly patients. GPs were motivated by positive preventive experiences, but often lacked the necessary training to counsel and support their patients.

**Conclusions:**

German GPs had positive attitudes towards prevention, but the implementation of preventive care was neither systematic nor continuous. Identification and elimination of barriers to preventive care is crucial. Further research is needed to identify effective practice-based approaches to overcome these barriers.

## Background

General practitioners (GPs) have a pivotal role in the delivery of preventive care, particularly for the elderly. In 1996, the US Preventive Services Task Force concluded that effective primary preventive care was more effective in improving health than many of the routine examinations used for early detection of diseases. A few years later, additional evidence on primary preventive care confirmed this conclusion [1]. At the beginning of this decade, few studies had examined the effect of medical counseling [2], but such studies are becoming more common [3,4].

Recent studies have identified barriers to the implementation of primary prevention. In particular, there are several proposed frameworks that classified the barriers as being associated with users, physicians, practice organizations, healthcare systems, and the specific preventive guidelines [5,6]. Additional studies have identified a discordance between the positive attitude of GPs regarding primary prevention and the actual implementation of preventive care [7]. Even in cases where GPs implement primary prevention, it is often inadequate [8]. Thus, the daily primary care practice, which is dominated by diagnoses, treatment of acute health problems, and secondary prevention, is a significant barrier to the implementation of primary preventive care [9]. Additionally, many GPs consider secondary prevention as more effective than primary prevention [10].

A previous study of cardiovascular disease (CVD) prevention showed that GPs underestimated the risk of CVD. More than half of the surveyed GPs did not use any comprehensive tool for global risk assessment of CVD [11]. With regard to tobacco smoking, most physicians saw it as their duty to discuss and counsel patients on their smoking habits, but few physicians asked about unhealthy habits before the symptoms became manifest. Moreover, few physicians provided patients with adequate advice on smoking cessation. The time-consuming nature and low effectiveness of smoking cessation programs may be considered barriers to the implementation of this specific preventive therapy [12]. A recent study compared GPs of the US, UK, and Germany using videotaped patient consultations, and reported that German GPs gave less preventive advice than GPs from the UK and US. Additionally, in Germany there were significant differences in the preventive consultations given to older patients (> 75 years) and younger patients (< 55 years) [13,14].

A European study on the implementation of clinical practice guidelines for the prevention of coronary heart diseases identified the most common barriers to implementation as lack of time, prescription costs, and patient noncompliance [15]. A Swiss study reported similar results regarding alcohol and nutrition counseling interventions [16]. Studies of physicians and nurses reported lack of motivation, inadequate reimbursement, and discontinuity of care as barriers to the implementation of preventive guidelines [17,18]. A European study of 11 countries (excluding Germany) reported that more than half of surveyed GPs were skeptical about the effectiveness of counseling that is designed to help patients change unhealthy behaviors and that there was also an association between a GP's own personal health-related behaviors and his/her attitudes toward health promotion and prevention, the only exception being in the case of obese GPs who advised overweight patients [19]. Additional barriers to preventive care include physical and mental co-morbidities of patients [15] and lack of knowledge about the advantages and difficulties of integrating preventive activities into everyday life [20].

Changing the reimbursement system, continuous improvement of medical practice, use of modern technologies for management of patient data, and availability of well-trained staff are among the most important factors that contribute to better implementation of preventive intervention in practice [21]. Additionally, group practices offer more preventive services than GPs who practice alone [4].

Thus, to encourage physicians to deliver adequate preventive care it is essential to recognize the needs and opportunities for integration of behavioral counseling into medical practice [16,21,22]. This is particularly important owing to the increasing proportion of older patients and the importance of preventive measures for older patients. This article examines factors that inhibit and those that promote the implementation of preventive care in the daily practice of German GPs, particularly for elderly patients.

The theoretical context of the present study is based on Social Representations Theory [23,24]. This theory deals with the integration of scientific concepts into everyday knowledge and practices ("anchoring") and objects or images to which an abstract process is linked ("objectification"). One concern is how these representations differ among social groups, such as GPs and nurses. In contrast to attitudinal research, which focuses on individual knowledge, social representations are considered to be "social knowledge". Moscovici considers attitudes as one dimension of social representation [24].

The present study examines factors that promote and those that inhibit the implementation of preventive care for the elderly, as perceived by GPs in Berlin and Hannover, and also examines changes in physician perception of promoting and inhibiting factors over the course of their careers. These results are drawn from a study the main objectives of which were to determine how GPs understand prevention and health promotion for their elderly patients and how they view aging.

## Methods

The study "Perception of health and age by physicians and home care nurses" was conducted between 2001 and 2003. We obtained ethical approval from the Research Ethics Committee of Hannover Medical School. Our aim was to have a sample size of 32 GPs, 16 in Berlin (B) and 16 in Hannover (H), which would enable generalization of our results to all German GPs in general practice.

In Hannover, according to the register of the Medical Associations and Regional Associations of Statutory Health Insurance (SHI)-Accredited Physicians, there were 332 physicians (including internists, general physicians, and practicing physicians) working in the city in 2001. In Berlin, the publication "Medicine in Berlin 2000", which lists all physicians working there, and the register of the Association of German Internists were used to select all internists, general and practicing physicians (N = 320) in the Neukoelln and Steglitz/Zellendorf districts.

In each city, a total of 100 eligible GPs were contacted by letter. Inclusion criteria were: minimum professional experience of 5 years, age 40-50 years, and employment in underprivileged or middle-class areas, and employment as a practicing physician, internist, or physician working in general medicine. The study consisted of two groups of 32 registered GPs and 32 home-care nurses in the two cities; data from home-care nurses is not included in the present study. All participating GPs provided informed consent and were paid 50 € as an honorarium.

This explorative study used episodic interviews. The episodic interview is a special narrative interviewing method of data collection that elicits descriptions of particular features or processes in the interviewee's daily life. The objective of episodic interviewing is to facilitate and encourage the sharing of anecdotes that may be particularly important and rich with detailed information about daily experiences of the participants [25].

An interview guide was developed and pre-tested with six physicians in 2001. The pre-test was carefully analyzed and detailed information about the following was reviewed: clarity and design of the questions, whether the questions elicited the desired information, and whether the participants understood the questions. Further data about the interviewee was also documented. The pre-test and the actual interviews were performed by two well-trained sociologists. Furthermore, because people differ in their natural ability to provide anecdotes, the interviewer explained the rationale of the episodic interview so that participants would be encouraged to provide anecdotes that were as detailed as possible.

The interview had 15 questions in three main blocks. The first block focused on the image of the aging of elderly patients as seen by the GPs. The second block focused on the importance and chances for prevention and health promotion for elderly people. The third block was a combination of both concepts. Additional questions concerned actual situations that the physicians experienced.

A central block of questions was devoted to prevention and health promotion to determine GPs understanding and implementation of prevention and health promotion activities in their daily practices. GPs were not directly asked about inhibiting and promoting factors. Instead, these were inferred from their responses to the question: "What is the relevance of prevention and health promotion (for elderly people) in your professional practice? Please describe a situation for me that demonstrates it". Further questions included "Please tell me, how your day went yesterday and how, when and where health promotion played a role in it" and "Did your professional work change in recent years concerning the promotion of health? Please describe a situation for me that shows these changes".

Socio-demographic and structural data of the GPs were documented in a short questionnaire that collected information about the GP's family status, specialty, age, gender, and details about the interview (e.g. duration, time, and place). Detailed information about the content of interviews, including remarks, characteristics, and special features of the interview that occurred after the video-recording stopped were also documented.

All interviews were followed by a video-assisted interview training session and were tape recorded and transcribed, with thematic coding used for data analysis. Codes were developed from the interview transcripts and a short description for each case, which included key statements, was produced to facilitate comparative analysis of materials using the computer program ATLAS/ti [26]. This program facilitates separation of text into paragraphs, graphical illustration of the associations between different codes and categories, and discrete management of codes and memos, such as notes for theoretical and methodical parts of the interview. After interview analysis, communicative validations of the data were shown to the GPs, who were asked to consent, reject, or correct the findings. All participating GPs who were interested in further discussions were invited to participate in focus group discussions. Seven GPs participated in these focus groups and the discussions involved presentation of the results of interviews and discussion of the identified barriers to implementation of preventive measures and possible practical solutions to overcome these barriers. The findings of the focus groups are not covered in the present paper.

## Results

We aimed to interview 32 GPs, but ultimately conducted 36 interviews because four interviews were not recorded due to technical problems. The data from all 32 complete interviews were analyzed.

The average interview lasted 53 minutes (range 20-89 min). Two-thirds of the GPs were male (n = 21). The average age was 48 years (range 39-59 yrs). Adherence to one of the inclusion criteria (age between 40-50 years) was not maintained because seven GPs were 51-59 years old. On average, the women (51 years) were older than the men (47 years). The participants had been in practice for an average of 8.5 years. The female physicians had more experience (max. 24 years) than the male physicians (max. 21 years). None of the GPs had attended training courses or supplemental education programs in geriatrics. About one third of the participants had practice populations in which 51-60% of the patients were aged more than 60 years.

In general, no GPs categorically rejected the idea of behavioral prevention. But in the interviews, they identified more inhibitors than promoters of preventive care. Only three of the interviewed GPs recognized no barriers to prevention. The remaining 29 physicians described more than 90 barriers, indicating a dissatisfaction with or difficulty in implementing prevention methods. Only 14 physicians identified promoting factors for prevention, yet they did not extensively describe these factors. Only 13 physicians mentioned specific barriers to prevention for elderly patients, and most of these were healthcare system-related barriers. In old age, only six GPs reported promoting factors for prevention. Table 1 summarizes the main inhibiting and promoting factors perceived by GPs.

**Table 1:** Factors that inhibit or promote preventive care from the GP's perspective.

Inhibiting factors related to	Promoting factors related to
***Patients***	***Patients***

Patient attitude: no motivation, (patients want to be left alone, resistance, no candidness, inflexibility, negative attitudes, passive expectations, prevention is not possible in geriatric cases)*	Patient attitudes: motivation, increasing demand
Patient characteristics: low literacy, age*	Information from internet and media
Unnoticeable risk factors and repression of unpleasant findings	Crisis health situation
Difficulty implementing behavior changes	Willingness to pay additional costs*
No willingness to pay additional costs	
No support from family*	

***GPs***	***GPs***

GP attitudes: negative attitudes and own health habits, low motivation for counseling, (restraining commitment and motivation of patients by GPs)*	Positive experiences
Financial concerns	Spending time aimed at increasing compliance and motivation
Lack of time	Positive resonance through preventive offers, e.g. courses
Focus on acute care	Financial support*
Lack of persuasion ability	
Lack of awareness of preventive measures for elderly*	

***Healthcare System***	***Healthcare System***

Acute-care orientation of health system	Health promotion is a huge field of investment
Absence of political will to invest in prevention	More offers for preventive care being a new topic in the media
No/inadequate reimbursement	Health insurance companies are obliged to financially support preventive care
Limited number of offers from health insurance companies	No facilitators for elderly patients were mentioned
Fragmentation of care	
No social interest in preventive care in old age*	

* Additional determinants for preventive care in elderly individuals from the GP's perspective

### Patient and physician related inhibiting factors

Patient attitude was an important determinant of a physician's acceptance and utilization of preventive care. According to the GPs, many patients were not motivated and were unwilling to change their unhealthy behaviors. Despite suggestions that patients could do something to prevent illnesses, many patients seemed uninterested in making lifestyle changes. The physicians believed that nothing was to be gained if the initiative only came from the physician.

Lack of patient motivation often occurs because some risk factors, such as hypertension and high cholesterol levels, have no symptoms. Moreover, many patients tended to repress unpleasant medical findings. In fact, there was often a discrepancy between the patient's self-perception and the medical diagnostic findings. "Behavior modification is difficult, particularly for those who do not feel ill or who are not aware of their health problems. (...) The symptoms come later anyway" (GPB14). Therefore, physicians viewed crisis situations as an opportunity to modify risky health-related behaviors. Here, physicians saw it as their specific duty to help patients change unhealthy behaviors. "It is my duty to advise patients on their unhealthy lifestyles. If you convince only one person out of a 100 patients in a day, and they successfully achieve a permanent change, then I have really accomplished a lot for one day" (GPH05).

Some physicians stated that they were aware of the difficulties patients had in making changes in lifestyle and reported that many patients did make efforts to change their unhealthy behaviors, but they simply were unsuccessful. This may be due to a difficulty in implementing a physician's specific recommendations because the patient did not really want to change, or because the patient is psychologically incapable or unprepared for change. The perceived unwillingness of patients to change unhealthy lifestyles leads many physicians to avoid preventive care: "When I counsel patients who smoke two packs a day for a few minutes on tobacco use, and advise them to quit, I notice their unwillingness to change their behaviors. Then my motivation for prevention is very low" (GPH05).

Physicians' own health-related habits and their attitudes towards prevention were important determinants of their delivery of preventive care. GPs recommended preventative measures less often or with less conviction if they did not practice preventive measures themselves. In part, these physicians seemed unsure about how they would react to prevention measures that required lifestyle changes.

Physicians also expressed frustration with their inability to reach all population groups, such as those with low literacy. Also, GPs did not target healthy people, and most did not consider the "healthy segment" of their own patients; "If you have a healthy patient in front of you who does not have any risk factors and also does lots of sport, you can't do anything for him. That's ok and you should tell him that" (HGP18). Just four GPs recommended that their patients who did not have risk factors or diseases should do some exercise. Therefore, GPs tended to downplay their roles in primary prevention and health promotion.

### Patient and physician related promoting factors

Physicians reported some success in promoting factors, such as getting a patient to quit smoking or getting a patient to adhere to a weight management program or physical activity course. One GP, who normally had a skeptical attitude about prevention, talked about an example of successful prevention in a 65-year old woman who was previously a heavy smoker and had a very high cholesterol level: "She stopped after I told her again clearly what would happen [...]. I myself was astonished when she told me that after only a few weeks she had stopped smoking and now only needs medication to keep her blood pressure down, which she has got used to very well, has lost weight and has even enrolled at a gym. That is definitely a case where I would say that preventative guidance has at least helped" (HGP04). Positive remarks came from two physicians who explicitly stated that they enjoyed using preventative measures.

The GPs identified certain behaviors and experiences that increased their use of preventive care, including health check-ups, vaccinations, understanding specific health concerns and health education needs of their patients, and allowing patients the time to thoroughly explain their health problems. Several GPs also emphasized that patients increasingly understood the importance of prevention due to media reports about risky behaviors, diseases, and health promotion. Increased demand for preventive care was an important promoting factor.

### Prevention in old age

Physicians also perceived advanced age as an obstacle to health promotion because they perceived elderly people as more resistant to change and believed that prevention was not possible for geriatric patients. They also believed that integration of everyday lifestyle changes following hospitalization were not sustainable. The GPs stated that their elderly patients had negative attitudes towards prevention or had minimal expectations about the efficacy of prevention and behavioral change. In this regard, GPs stated that patients often do not work on their own to change their behaviors, but expect their physicians to do the work.

On the other hand, we identified two obstacles on the part of physicians: ***(i) ***lack of awareness of preventive measures for elderly patients; and ***(ii) ***lack of motivational counseling skills and experience. Nevertheless, some doctors emphasized that prevention, especially exercise, was precisely what elderly people and elderly patients often required: "The older a person is, the more important health promotion and prophylactic medicine naturally become" (BGP02). "Then the patient asks: 'Is it worth it at this age, at my age?' Naturally, we could agree with them, but we can then say that it would be nice to make life worth living in the last year, 2 years of their life by improving the quality of life a little" (BGP07). From his own experience, one doctor emphasized the importance of maintaining mobility in elderly people: "If you take everything from a patient, especially the older ones, they will become even more immobile and dependent. It's imperative to give them challenges" (BGP15). Two doctors stressed that health promotion and prevention is "completely unrelated to one's age".

Experience with elderly patients who were still active, highly motivated, and always had healthy lifestyles tended to motivate GPs to offer preventive care: "It is interesting to listen to those elderly people talking about their lifestyles. They do various activities to remain healthy. They train their mental capacities by reading or attending theatre events or traveling. Riding bicycles, walking, and other types of sports are in their daily program. Generally, those patients have always led healthy lifestyles" (GPH18). Additionally, a patient's willingness to pay additional money for specific preventive offers was considered a promoter of preventive measures in old age.

GPs with more negative attitudes about prevention attached greater importance to prevention for young patients: "For me, prevention is above all for 40 and 50 year olds, and perhaps for those who are younger, those who have 20 or 30 years in front of them, where you want to change and avoid something tangible" (HGP12). Some failed to see any preventative potential for elderly individuals. This was reflected in remarks such as: "It's basically too late for the elderly. You can't help them much more" (BGP15); and "You won't find much prevention in geriatric medicine" (BGP12). Other GPs regarded prevention in old age as being unimportant: "The things that are good for you are also good for them. Therefore, eating less and more physical exercise are naturally of extraordinary benefit - they don't have to take up sport, it's about taking walks, every day if possible. [...] Older people should also keep physically fit and eat healthily, why not? What I mean though, is that it's not so imperative or important as at a younger age" (BGP08). At the same time, physicians seemed to support their patients: "Health prevention doesn't play a role anymore. They always say: 'just leave me be, I'm already so old.' When it comes down to risk factors, they are right. I'm quite broad-minded about it" (BGP08).

Frequently, GPs differentiated among their elderly patients. While some regarded prevention as beneficial for those aged 60-70 years, some regarded prevention as useless for those who were older, especially for those suffering from age-related problems: "For octogenarians, nothing more has to be done. What's the point? If they are super-fit and want to do something, ok, but otherwise it doesn't make much sense" (HGP13). Correspondingly, the GPs believed that laboratory measurements (such as blood pressure, and cholesterol levels) were less important for elderly patients: "Of course, less and less prevention in old age. In the last years, you won't find much prevention. There aren't many possibilities for prevention here. I would therefore rather reduce tablets, or even stop them in old age, like for cholesterol prophylactics for instance, give them less medication. And I'm more generous when it comes to blood pressure and sugar levels too. Here, other criteria have more importance than those in guidelines or standards" (BGP11).

Two GPs noted that only secondary and tertiary prevention were possible for elderly patients due to the restrictions already experienced by many of these patients. They considered their main aims to be the sustaining of current functional capabilities and the prevention of further restrictions. When GPs were asked to precisely define prevention for the elderly, they said that the maintenance of independence and mobility "plays a more important role than medication" (BGP11). It is striking that some GPs described preventative measures in old age as "little", "simple" and "banal things", as well as "not anything over the top". For example, "In old age, it was about [...] getting people to do really simple things that don't cost anything, like going for a walk every day" (HGP19).

### Healthcare system related factors

Statements concerning the role of the healthcare system in the implementation of preventive care were similar. Most GPs referred to the prioritization of acute care and non-preventative measures in the current healthcare system. The GPs also noted that the reasons for this were due to a lack of interest in prevention by the pharmaceutical industry, their colleagues and the entire healthcare system, and the absence of political will to promote prevention. At the sociological level, GPs complained about a lack of effective healthcare campaigns targeting tobacco smoking and legal obligations. The absence of interest in prevention for the elderly was also mentioned: "Preventive activities that help promote health and prevention are also reasonable for older people. Yet, those activities are very expensive, so the value is placed somewhere else, and it is not important if elderly people live healthy, happy and satisfied lives. This is because they are not productive anymore, thus there is no social interest to invest in this field, it is a problem related to our society" (BA09, 1149-1166).

GPs complained that preventive services, including primary prevention and counseling interventions, were not adequately reimbursed and sometimes not reimbursed at all. They were concerned that they would earn less if they administered preventive care: "But may be the reason is that we earn less, if so many check-up measures are applied. Higher reimbursement rates exist for treatment of, for example, acute health problems, than for prevention" (GPB01).

Other important barriers to the implementation of preventive care were the lack of available preventive services in the community, lack of knowledge about the preventive services that were available, discontinuation of care by healthcare providers, and long waiting time for the existing offers from the health insurance companies, e.g. cooking courses. The GPs identified few factors in the German healthcare system that promoted preventive care, but some physicians emphasized the support provided by sickness funds: "But, it is also quite interesting to see that the sickness funds are of course required by law to spend money on health-promoting activities, and, since then, it has become a much larger industry. There are certainly many effective and reasonable offers including courses for low-back pains, sport courses, and nutrition educational programs, etc." (GPH17).

### Transition

This term refers to a physician's perceptions of his/her own changes in attitude regarding preventive care over the years. It provides an indication of changes in promoting and inhibiting factors related to the delivery of preventive care. Below, we discuss these as "personal changes" and "social health policy changes".

### Personal change

Twenty-six GPs provided statements concerning personal transitions throughout their careers regarding their attitudes towards prevention or the implementation of preventive interventions. Twelve physicians noted that they developed more positive attitudes toward preventive care over time.

Working in ambulatory healthcare settings and practical experience led GPs to place increased importance on prevention. In ambulatory healthcare settings, in contrast to work in the hospitals where "thinking only as far as the release of the patient" prevailed, physicians got to know and observe their patients more closely. Increased practical experience and better knowledge of the patients gained through their many years of work also promoted the use of systematic preventive interventions. Some GPs indicated that their emphasis on the relative importance of prevention had increased over time. They placed greater value on prevention, healthy nutrition, good physical fitness, and the avoidance of drug use because they saw many cases where diseases could have been prevented if risky behaviors had changed. Other physicians emphasized that, within the course of their careers, they had come to appreciate the importance of telling patients that they were responsible for their own health-related behaviors.

On the other hand, several physicians indicated that they developed more negative attitudes toward prevention over time because their past efforts had achieved limited or no success. Nine physicians indicated they had no personal changes regarding prevention in recent years; "The receptiveness and the capability of patients to put the recommendations into practice are limited. This is particularly true for nutritional problems. You hope that patients would permanently implement a minimum of the physicians' suggestions. Well, I think I have used the conjunctive case three times, and that is exactly how I meant that. And, if you want to convince patients that this change of diet is absolutely necessary, and you can assume that they have understood it, then by no means does that mean that they will make a change someday. Or that they will make a change for 1 day, for 1 week, 1 month, a year, or for the rest of their life. This is the reason behind scaling down my expectations and realistic estimations to a minimal level, for the practical and permanent implementation that can be achieved in one year" (GPB08). Currently, this GP provides prevention counseling to patients who seem willing to change their unhealthy behaviors. This approach was also used by four other participating GPs; "What has changed is that I select individual patients in a more systematic manner. Whereby, I particularly support those persons who have had a heart attack and no longer persons with risk factor. I will certainly address the matter, but I will no longer invest my energy if I notice that the patient is not willing to change his behavior" (GPB11).

### Social and health policy transition

Six physicians indicated that the social and political conditions that allow for preventive care have improved. They noted an increasing demand by patients, an increasing range of available preventive services, and improved offers and information about prevention. In the past 20 years, health promotion has become common and is widely covered in the media. As noted by one GP, "I am offering more preventive interventions. Also because I notice that there is a great demand for prevention. Well, I am expanding prevention measures by making it more public. We have designed a brochure in the office, in which prevention has a significant role. Additionally, prevention is also presented on our website" (GPB11).

Four GPs noted a deterioration of factors that promoted preventive care and an increase in barriers, such as limited subsidies, for the implementation of such measures. One GP indicated that billing problems and other aspects of the healthcare system have led him to reduce preventive care in his office.

## Discussion

This study identified many factors that inhibit and many factors that promote the implementation of preventive care during general medical practice in Germany. In our study, these factors were classified as related to patients, physicians, or the healthcare system. Previous frameworks identified additional types of barriers, such as the practice organization [6] and medical recommendations [5]. Inhibitory factors were mostly viewed as originating from the patient, but the healthcare system itself was considered to insufficiently promote preventive care. Factors that promoted preventive care in the elderly were mentioned by less than one-fifth of participants. Similar results have been reported previously [5].

GPs placed great importance on their patients' attitudes toward preventive care. Previous studies of preventive care have reported that there was a lack of patient motivation and willingness to change, and low compliance with physicians' recommendations [9,27,28]. Furthermore, GPs expressed concern about reaching and counseling specific high-risk groups, such as patients with poor literacy.

Our study participants indicated that their lack of willingness to pay for preventive care that is not covered by insurance companies was a major barrier. The type, intensity, and costs of those preventive measures vary among physicians; however, a range of costs is listed in the fee schedule for physicians. Additionally, some of those services are not supported by evidence-based medicine. For example, whether the costs for a glaucoma examination are covered depends on the necessity of the test for each individual patient. We suggest that patients ask their physicians about advantages, necessity, and reliability of specific preventive care tests [29,30].

In this study, GPs stated that their motivation for providing preventive care was lower for patients who had poor compliance. Hudon et al. reported that the lack of physicians' motivation for preventive counseling was an important barrier in the integration of clinical guidelines into daily practice [5]. Cohen et al. suggested using the transtheoretical model to increase physicians' motivation for providing preventive care. In particular, they suggested implementing a whole-system strategy that targets physicians, staff, and patients. This strategy includes various interventions, such as support from medical institutions, chart flow sheets, practice feedback reports, and designated preventive care coordinators [31].

Time constraints due to heavy workloads contribute to the delivery of less preventive care in the general medical practices of Germany. A 2007 study compared primary care physicians in seven countries (including Germany) with regard to their daily workloads, quality of healthcare services, and satisfaction with the healthcare systems. In Germany, GPs had the greatest number of patients per week (N = 243). For the other 6 countries, this number ranged from 102 patients (USA) to 154 patients (UK). German physicians also had the greatest number of work hours each week (N = 51 hours). The heavy workload of German physicians resulted in less time allocated for each patient (less than 8 min in Germany, but between 13 and 19 minutes in five of the seven other countries). This in turn led to problems regarding the quality of care [32]. The results of this study are in broad agreement with other studies that reported that a German physician in the ambulatory health sector has about 225 patients per week [33,34]. Generally, German patients visit their GPs more often than patients in other countries [33,35]. According to a report of the *Gmünder Ersatzkasse *that was based on invoicing data, 68.0% of insured patients had at least one contact with their GP in 2007 [36]. A patient's average number of annual visits to a physician increases considerably with age, and was also increased in 2007 compared with 2004. In 2007, the average 45-year old German man visited his GP six times, and the average 45-year old German woman visited her GP eight times [33,36]. This high frequency of contacts provides the GP with ample opportunity to act as a health educator and counselor, which is particularly important for elderly people.

Delegating some of the preventive care activities to nurses and other qualified healthcare personnel may help to resolve some of the problems related to the heavy workload of German GPs. Many previous studies have investigated the effectiveness of behavioral counseling by nurses. Other members of the health team, such as nurses and healthcare educators, can help to manage patients by contacting and supporting them, in addition to coordinating care with other organizations outside their own practices [2,17]. However, a study in Germany reported that less than 50% of participating GPs thought that a brief counseling intervention that targeted tobacco smoking could be performed by other qualified medical staff [18]. It would be interesting to investigate the reasons behind this attitude, and to ask GPs about alternatives for improving the delivery of primary preventive care in Germany.

In the present study, some of the GPs recognized that their own healthcare habits and attitudes towards prevention could influence the delivery of preventive care. For example, physicians who consumed more than three alcoholic drinks per day, had sedentary lifestyles, or were unaware of their own blood pressure were more likely to have negative attitudes about preventive care [16]. This in turn made them less likely to recommend preventive healthcare measures, or to place less emphasis on such interventions. In addition, GPs may not feel comfortable in providing behavioral counseling, due to their lack of communication skills. Previous studies have also reported this particular problem [6,27,28]. Regarding preventive care for the elderly, none of the GPs in our study had attended a training program or had further education in geriatrics. Pham et al. reported that physicians with additional qualifications and training in their primary fields were more likely to deliver preventive services to the elderly [4].

The lack of training and the low level of trust in the effectiveness of the physician's own counseling abilities aggravate the perceived barriers to the implementation of preventive care [9,37]. Identification of patients at the greatest risk and those most willing to change according to the transtheoretical model is considered to be important for the appropriate provision of preventive measures. This is particularly important in the field of non-primary-care medicine, where physicians may not feel competent to provide preventive care. Previous studies have indicated that physicians were aware of their own needs for training in behavioral management strategies, approaches for successful discussion of management, and in addressing family conflicts and motivating patients [17,38,39].

Barriers to preventive care in the healthcare system include the emphasis on acute-care and inadequate or no reimbursement for preventive-care interventions. In this regard, it is necessary to mention that in Germany, two paragraphs (§ 20 and § 25) in the Social Security Code specifically refer to preventive interventions [40]. Paragraph 20 states that health insurance companies should offer primary preventive services that promote health status and decrease social inequities, although it does not specify the type or intensity of the interventions. Preventive services vary among insurance companies, and may include courses and setting-specific programs in schools and companies. The fee allocated for primary preventive measures was 4.83 Euro per insured individual per year in 2009 [41]. In 2007, only 4.2% of insured persons of the SHI used these services. SHI companies developed a bonus system for their patients, in which patients were given monetary incentives or small presents for using primary preventive services, such as health check-ups, oral prophylaxis, and vaccinations. Paragraph 25 refers to various preventive services, such as cancer screening (breast, prostate, and colon) and a check-up after patients are aged 35 years, which are reimbursed expenses. There are no budgetary limitations for those services. The check-up includes tests for early detection of diabetes mellitus, cardiovascular disease, and kidney disease [42,43]. Preventive care for the elderly includes examinations for functional disabilities and consideration of cardiopulmonary and neuromuscular status, which are also reimbursed by SHI companies [40]. However, there are still no systematic primary preventive interventions by GPs in Germany.

Fragmentation of care and lack of cooperation among healthcare providers in the field of prevention and health promotion are also barriers to preventive care. Continuity of care and frequent patient-physician contact provide more opportunities for preventive care and allow GPs to more easily counsel patients and guide them through the process of lifestyle changes. Unfortunately, medical education in Germany has not given sufficient emphasis to preventive care. The results of the present study show that physicians recognize the importance of preventive care and have obtained knowledge about preventive care throughout their careers. In Germany, an obligatory course "Prevention and Health Promotion" was first introduced to medical training in 2003 [44] and continuing training programs for physicians were first introduced in 2004. The impact of those two programs on the perception and implementation of preventive care should be examined in future studies.

From the physicians' perspective, preventive care would be easier if they had the time to explain to patients the causes and consequences of their potential health problems. Our results indicate that GPs are motivated to provide preventive care if they have had positive experiences with previous patients. However, many GPs underestimate the importance of preventive care because only ill people visit them. They do not see the positive impact of preventive measures in healthy people. However, the health of a population can be more greatly improved by reducing or eliminating risk factors in the many people who are not considered high risk, than by reducing or eliminating risk factors in the few high-risk people [45,46]. This is hard to comprehend in medical practice with its concentration on individuals. Thus, the development and the introduction of systematic interventions for all patients that can be integrated in routine healthcare would be a significant advance. Examples of this are the "5 A" concept, the stages of change theory, and counseling instruments for quitting smoking and reducing alcohol consumption [2,21,47]. These approaches would give physicians more confidence in performing behavioral counseling [48]. When implemented, the current selective approach of physicians toward preventive care (only for health-conscious patients willing to change) and the perceived increasing demand for prevention from the patients can be counteracted in a systematic manner.

The support of healthcare policies and all healthcare providers, as well as adequate financial reimbursement, would help to improve the delivery of preventive services. There are many similarities between the German and Austrian healthcare systems with regard to financing and reimbursement; hence, it might be helpful to study the Austrian experience in overcoming barriers to the delivery of preventive care. In Austria, a new periodic health check-up guideline was introduced in 2005. This guideline targeted certain health problems such as cardiovascular disease, tobacco smoking, and periodontitis. Barriers to the implementation of this new guideline were overcome by increasing reimbursement fees up to 75€ for each examination and changing the documentation system. Another planned change, although not yet implemented, is a "reminder system" for GPs and their patients [49,50]. Many previous studies have demonstrated that use of computer-based reminder systems lead to greater use of preventive services [51,52].

Our study has several limitations. First, selection bias may have resulted from the non-random manner of selecting study participants. Second, the study was conducted in two large cities, which limits the extent to which the results can be generalized to rural areas. It would be interesting to replicate this study in one or more rural areas in Germany. Third, this study focused on a small group of GPs, but there are other healthcare professionals in the ambulatory healthcare sector whose beliefs and experiences are important to the provision of preventive care. However, this is the first study to examine the attitudes and daily practice of German GPs in providing preventive care.

## Conclusions

This study provides insight into the methods currently used to implement preventive care measures in general medical practices in Germany. We found that preventive care was provided in a suboptimal and unsystematic manner. The participants of this study learned about preventive care from their own experiences in their medical practices. Preventive care was not a major part of the medical curricula and there are limited continuous training programs offered by German health organizations. This hinders the delivery of systematic and continuous targeted preventive care, particularly to the elderly.

At present, well-tested and effective approaches for the delivery of primary preventive care to the elderly do not exist in Germany. Additionally, there is no nationwide implementation of well-established programs with this aim. Thus, knowledge gained through practical experience is considered essential for the provision of age-targeted preventive care and healthcare promotion activities. The problems seem to be in transferring the GPs' theoretical knowledge of preventive care into practice and the lack of self-confidence in the effectiveness and value of prevention.

The participating GPs identified barriers to preventive care mainly as originating from the healthcare system and patients. Elderly people should be informed about the benefits of preventive measures, perhaps by an initiative to change the German healthcare system. This change can be attained through a holistic approach, which can be initiated by the inclusion of primary preventive services in the health services catalogue of insurance companies for the elderly. For example, the Advisory Counsel on the Assessment of Developments in the Healthcare System could advise on preventive interventions for the elderly, thereby reaching the entire community [53].

## Competing interests

The authors declare that they have no competing interests.

## Authors' contributions

UW was responsible for designing the study, interpreting the findings, and writing the paper. UF was responsible for designing the study, interpretation, and writing the paper. AN was responsible for data collection, analysis and interpretation, and writing the paper.

CF was responsible for the collection and analysis of data, interpretation of results, and writing the paper. RJH was responsible for writing and revising the paper critically for important intellectual content. FWS was responsible for designing the study, interpreting the findings, and commenting on drafts of the paper. All authors read and approved the final manuscript.

## Pre-publication history

The pre-publication history for this paper can be accessed here:

http://www.biomedcentral.com/1471-2296/11/68/prepub
